# Evaluation of the quality of care in pediatric hospitals in the Gaza Strip using the WHO integrated tool: the healthcare providers' perspective

**DOI:** 10.3389/fped.2025.1589092

**Published:** 2025-04-30

**Authors:** Saeed Y. Eleyyan, Bothyna B. ELssyed Etewa, Fatma Al’Haj Ahmad, Abdel Hamid El Bilbeisi

**Affiliations:** ^1^ Department of Pediatric Nursing, School of Nursing, University of Albutana, Rufaa, Sudan; ^2^ Department of Clinical Nutrition, Faculty of Applied Medical Sciences, Al Azhar University of Gaza, Gaza Strip, Palestine; ^3^ Department of Nutrition, School of Medicine and Health Sciences, University of Palestine, Gaza Strip, Palestine

**Keywords:** assessment, children, Gaza Strip, pediatric hospitals, quality of care, WHO

## Abstract

**Background:**

This study aimed to assess the quality of pediatric care in Gaza Strip hospitals using the World Health Organization (WHO) integrated tool.

**Method:**

A cross-sectional study was conducted in 2023 before Gaza war, with a census sample of healthcare providers at three major governmental pediatric hospitals: Al-Nasr, Al-Durra, and Al-Rantisi. Data were collected through an interview-based questionnaire, and statistical analysis was performed using SPSS version 26.

**Results:**

A total of 336 participants were included (59.5% male, 40.5% female). Of these, 13% were pediatricians, 16% general doctors, 1% pediatric nurses, and 70% were general nurses. The overall total scores for healthcare providers' responses to the eight WHO quality standards was 29.5%; in addition, it was varied across hospitals: Al-Nasr (29.1%), EL-Rantisi (31.9%), and Al-Durra (28.4%). The lowest score was for Standard VIII (8.3%), related to the physical environment, while the highest score was for Standard V (70.9%) related to rights of children. Statistically significant differences were found for standards I, II, IV, VII, and VIII between the hospitals (*P*-values < 0.05 for all). Additionally, the health care providers aged 31–35 years were three times more likely to have better overall total scores compared to other age groups [Adjusted Odds Ratio = 3.014, 95% Confidence Interval = (1.255–7.241), *P* value = 0.014].

**Conclusion:**

The study revealed suboptimal pediatric care quality in Gaza Strip hospitals. The lowest scores for the healthcare providers, was 8.3% for standard (VIII), which pertains to the physical environment, emphasizing the need for infrastructural improvements, while the highest scores 70.9% was for standard (V) shows the providers respectful view and acts for children rights without discrimination. There is a need to prioritize upgrading the physical environment and ensuring the availability of essential resources, such as water, sanitation, and medical supplies, to enhance the overall quality of care for children in these hospitals. This study provides valuable insights for policymakers and healthcare professionals working to improve pediatric care in Gaza.

## 1 Introduction

The quality of pediatric care is a critical determinant of health outcomes for children, influencing both their short-term recovery and long-term well-being (1). Globally, pediatric care faces numerous challenges, including resource limitations, workforce shortages, and gaps in specialized care (2). In conflict zones, such as the Gaza Strip, these challenges are magnified due to the ongoing socio-political instability and limited access to health resources (3). Quality healthcare for children in the Gaza Strip is essential, not only for addressing immediate health needs but also for promoting sustainable development in the region (4). The World Health Organization (WHO) has developed an integrated tool for improving the quality of pediatric care in health facilities, designed to assist healthcare providers in delivering comprehensive, evidence-based care. This tool assesses various domains of care, including evidence-based illness management, health information systems, referral efficiency, effective communication with families, staff competency, and the adequacy of the physical environment (5).

In Gaza, pediatric healthcare services are provided by several governmental hospitals, including Al-Nasr Pediatric Hospital, Al-Durra Pediatric Hospital, and Al-Rantisi Pediatric Hospital. These institutions serve a crucial role in managing childhood illnesses and providing care to children up to 12 years old (6). Despite the critical nature of their services, little is known about the overall quality of pediatric care in these facilities. To the best of our knowledge, the healthcare providers' perspectives on the quality of care they deliver remain underexplored, particularly within the context of the WHO's integrated tool for pediatric care. Therefore, assessing healthcare providers' perceptions of quality care in the Gaza Strip is crucial for identifying strengths, gaps, and areas for improvement within these hospitals. Previous studies on pediatric care in the Middle East highlight several factors influencing the quality of care, including access to trained personnel, availability of medical supplies, and the physical infrastructure of healthcare facilities (7, 8). For instance, a study by Sami et al. (9) examined the challenges faced by pediatric hospitals in conflict-affected regions, noting that limited resources and overcrowded facilities were major barriers to providing high-quality care. Similarly, a study by Leary et al. (10) found that inadequate training for healthcare providers and insufficient pediatric-specific knowledge negatively impacted the quality of care in public hospitals.

The WHO tool is an essential resource for improving pediatric care, as it provides a comprehensive framework for evaluating the quality of care across different domains, including the physical environment, communication with families, and the competence of healthcare providers (5). However, the application of this tool in conflict zones, such as the Gaza Strip, requires particular attention to contextual factors, including ongoing shortages in medical staff, limited access to resources, and the political climate. The purpose of this study is to evaluate the quality of pediatric care in the Gaza Strip through the lens of healthcare providers' perspectives, using the WHO integrated tool as a framework. By assessing the quality of care provided in pediatric hospitals, this study aims to identify strengths and areas for improvement in these institutions. Furthermore, it seeks to understand the challenges faced by healthcare providers in delivering high-quality care under difficult circumstances. Ultimately, the findings of this research will serve as a valuable resource for policymakers, healthcare professionals, and planners, providing insights into the necessary steps to improve pediatric care in the region.

## 2 Materials and methods

### 2.1 Study design

This research is an observational, descriptive, and analytical cross-sectional study designed to assess healthcare providers' perspectives on the quality of pediatric care in Gaza hospitals, focusing on the integration of evidence-based practices, illness management, health information systems, and referral efficiency.

### 2.2 Study location and period

The current study was conducted in 2023 before Gaza war, in three major governmental pediatric hospitals in the Gaza Strip: Al-Nasr Pediatric Hospital, Al-Durra Pediatric Hospital, and Al-Rantisi Specialized Pediatric Hospital.

(1) Al-Nasr Pediatric Hospital: Established in 1962, it is the oldest pediatric hospital in Gaza, providing secondary healthcare services for children up to 12 years old. The hospital has 292 staff members and 121 beds, offering emergency, pediatric, intensive care, and neonatal services; (2) Al-Durra Pediatric Hospital: Opened in 2000, this hospital provides emergency, admission, and specialized services for children. It has 140 staff and 87 beds, with a radiology department managing 1,000–1,800 referrals annually; and (3) EL-Rantisi Specialized Pediatric Hospital: Founded in 2003 and fully operational by 2006, it is a tertiary facility with 287 staff members. The hospital offers 56 beds for children and 30 for adults across 15 specialized departments, including radiology, handling about 7,800 radiology referrals annually (6).

### 2.3 Study population

The study included all healthcare providers, regardless of gender, working in the selected hospitals in the Gaza Strip, including pediatricians, general doctors, pediatric nurses, and general nurses, who met the inclusion criteria and were present during the study period. Healthcare providers employed for less than six months, volunteers, and those who chose not to participate were excluded from the study.

### 2.4 Sample size and sampling technique

All participants working in the three selected hospitals at the time of data collection and meeting the inclusion criteria were included using a census sampling method. A total of 402 healthcare providers (pediatricians, general doctors, pediatric nurses, and general nurses) were eligible, with 336 participating, resulting in a 94% response rate. Sixty-six healthcare providers either refused to participate or were absent during the data collection period.

### 2.5 Data collection

#### 2.5.1 Interview-based questionnaire

A structured, pre-tested, and validated questionnaire was employed to gather data from each participant. The survey consisted of two sections:

##### 2.5.1.1 Assessment of participant characteristics

Data including age, gender, job role, qualifications, specialized pediatric studies, pediatric training courses, years of experience, and training duration, among others, were collected using an interview-based questionnaire.

##### 2.5.1.2 Assessment of the quality of care

The WHO integrated tool, “Standards for Improving the Quality of Care for Children in Health Facilities” was used to assess the quality of care provided to children in pediatrics hospitals in the Gaza Strip. The WHO integrated tool is structured into eight domains that focus on ensuring comprehensive, high-quality care for children. These include providing evidence-based care and management of illness according to WHO guidelines, ensuring effective health information systems for data collection and analysis, and facilitating timely referrals for conditions beyond available resources. Effective communication with children and their families is emphasized, ensuring their meaningful participation and respecting their needs and preferences. The tool highlights the importance of safeguarding children's rights, offering educational, emotional, and psychosocial support tailored to their needs. It also stresses the need for competent, motivated staff to provide consistent care, as well as maintaining a child-friendly physical environment in healthcare facilities, equipped with adequate resources like water, sanitation, and medical supplies for routine care (5).

#### 2.5.2 Translation and validation of the questionnaire

The translation of the questionnaire followed a cross-cultural adaptation process, as outlined in established guidelines (11). A five-step procedure was employed: (1) forward translation into Arabic by two native Arabic-speaking translators, (2) back-translation into English by two native English-speaking translators, (3) review by an expert committee, (4) pre-testing, and (5) finalization of the Arabic version.

Face and content validity of the final Arabic draft were independently assessed by a panel of eight experts, including researchers, academics, healthcare professionals, a head nurse, and pediatric doctors. The Content Validity Index (CVI) was calculated to evaluate the relevance of the questionnaire items (12), with all items receiving scores above 0.82, indicating strong relevance. Minor revisions in wording and structure were made following a consensus among the primary author (SYE), two pediatricians, and a head nurse. Subsequently, the questionnaire was piloted among 20 eligible healthcare professionals. The pilot study demonstrated good internal consistency, with a Cronbach's alpha of 0.84.

### 2.6 Pilot study

A pilot study was carried out with 20 participants to evaluate the questionnaire and data collection methods. Feedback from the pilot study led to adjustments being made to the questionnaire to improve clarity and accuracy for the main study.

### 2.7 Data analysis

Statistical analysis was conducted using SPSS version 26. The data analysis process included defining variables, data entry, cleaning, and analysis. Continuous variables were expressed as means ± SD, while categorical variables were presented as percentages. The Chi-square test was applied to assess differences between categorical variables. Adjusted Odds Ratio and 95% Confidence Interval (CI) was calculated using multinomial logistic regression analysis. A *p*-value of less than 0.05 was considered statistically significant. The total scores of the health care provider's responses (agree and strongly agree) to the eight standards of quality of care was calculated based on the average of eight domains, and was calculated for each domain separately for the three hospitals in Gaza Strip.

## 3 Results

This study was conducted in the pediatric departments of three major hospitals in the Gaza Strip under the Palestinian Ministry of Health: Al-Nasr Pediatric Hospital, Al-Durra Pediatric Hospital, and Al-Rantisi Pediatric Hospital, which provide care for children up to 12 years old. The study included a total of 336 participants, with 59.5% males and 40.5% females, with an average age of 36.20 ± 8.73 years. The majority (71%) were nurses, while only 0.6% specialized as pediatric nurses. About 64.6% held a bachelor's degree, with significant differences noted between job classification and qualifications across the hospitals (*P*-value = 0.003). Most participants (83.3%) had not completed specialized pediatric studies, although 58.3% had attended pediatric care training courses. Furthermore, 23.2% had less than five years of experience, with an average of 12.6 ± 5.7 years of total work experience and 9 ± 4.8 years specifically in pediatric care. The average duration of training was 10.3 ± 13 weeks. Statistically significant differences were observed in both specialized studies in pediatric care and training duration across the hospitals (*P*-value = 0.002 and 0.020, respectively) (Table 1).

**Table 1:** Characteristics of the study participants by hospitals.

Variables	Total (*n* = 336)		AL-Nasser pediatric hospital (*n* = 175)		EL-Rantisi Pediatric hospital (*n* = 77)		Al-Durra pediatric hospital (*n* = 84)		*P* value
	100%		52%		23%		25%		
Age (Mean ± SD) (36.20 ± 8.73)									
20–25 years old	32	9.5%	21	12.00%	1	1.30%	10	11.90%	0.058
26–30 years old	73	21.7%	39	22.29%	17	22.08%	17	20.24%	
31–35 years old	76	22.6%	35	20.00%	21	27.27%	20	23.81%	
36–40 years old	70	20.8%	41	23.43%	19	24.68%	10	11.90%	
>40 years old	85	25.3%	39	22.29%	19	24.68%	27	32.14%	
Gender									
Male	200	59.5%	98	56.00%	43	55.84%	59	70.24%	0.058
Female	136	40.5%	77	44.00%	34	44.16%	25	29.76%	
Job classifications									
Pediatricians	44	13.1%	31	17.71%	2	2.60%	11	13.10%	0.003
General Doctors	54	16.1%	32	18.29%	6	7.79%	16	19.05%	
Pediatric Nurses	2	0.6%	1	0.57%	1	1.30%	0	0.00%	
General Nurses	236	70.2%	111	63.43%	68	88.31%	57	67.86%	
Qualification									
Diploma	48	14.3%	25	14.3%	8	10.4%	15	17.9%	0.003
Bachelor	217	64.6%	105	60.0%	58	75.3%	54	64.3%	
Master	47	14.0%	25	14.3%	10	13.0%	12	14.3%	
Ph.D.	24	7.1%	20	11.4%	1	1.3%	3	3.6%	
Special studies in pediatric care									
No	280	83.3%	134	76.6%	71	92.2%	75	89.3%	0.002
Yes	56	16.7%	41	23.4%	6	7.8%	9	10.7%	
Training courses in pediatric care									
No	140	41.7%	70	40.0%	28	36.4%	42	50.0%	0.175
Yes	196	58.3%	105	60.0%	49	63.6%	42	50.0%	
Years of experience (Mean ± SD) (12.6 ± 5.7)									
<5 years	78	23.2%	48	27.4%	12	15.6%	18	21.4%	0330
5–10 years	74	22.0%	37	21.1%	21	27.3%	16	19.0%	
11–15 years	86	25.6%	45	25.7%	21	27.3%	20	23.8%	
>15 years	98	29.2%	45	25.7%	23	29.9%	30	35.7%	
Years of experience in pediatric care (Mean ± SD) (9 ± 4.8)									
<5 years	98	29.2%	55	31.4%	22	28.6%	21	25.0%	0.569
5–10 years	79	23.5%	40	22.9%	21	27.3%	18	21.4%	
>10 years	159	47.3%	80	45.7%	34	44.2%	45	53.6%	
Training duration weeks									
None	129	38.4%	66	37.7%	28	36.4%	35	41.7%	0.020
<5 weeks	54	16.1%	35	20.0%	6	7.8%	13	15.5%	
5–15 weeks	63	18.8%	29	16.6%	24	31.2%	10	11.9%	
16–30 weeks	42	12.5%	16	9.1%	13	16.9%	13	15.5%	
31–45 weeks	18	5.4%	10	5.7%	2	2.6%	6	7.1%	
>45 weeks	30	8.9%	19	10.9%	4	5.2%	7	8.3%	

Continuous variables were expressed as means ± SD, while categorical variables were presented as percentages. The Chi-square test was used to examine differences in the prevalence of different categorical variable. *P* value ≤ 0.05 was considered as statistically significant.

Table 2 presents the findings on the healthcare providers' adherence to the WHO guidelines for pediatric care across eight standards. For Standard I (“Evidence-based care and illness management”), 66.7% of healthcare providers met the guidelines, with a significant difference across hospitals (*P* = 0.005). For Standard II (“Health information system ensuring timely data collection and analysis”), 63.7% of providers were compliant, with significant variation between hospitals (*P* = 0.002). Standard III (“Appropriate referrals for unmanageable conditions”) showed 65.2% adherence, but no significant hospital differences (*P* = 0.208). For Standard IV (“Effective communication with children and families”), 69.3% met the criteria, with a significant difference by hospital (*P* = 0.008). Standard V (“Respecting and fulfilling children's rights”) had 70.9% compliance, but no significant variation by hospital (*P* = 0.547). Standard VI (“Protecting children's rights”) showed 64.9% adherence, with no significant differences (*P* = 0.421). Standard VII (“Competent, motivated, empathetic staff”) had 64.6% compliance, with significant hospital differences (*P* = 0.034). Lastly, Standard VIII (“Child-friendly physical environment and necessary resources”) had the lowest adherence at 8.3%, with significant variation by hospital (*P* = 0.001).

**Table 2:** The health care provider's responses to the eight standards of quality of care for children at the three main pediatric hospitals in the Gaza Strip.

Variables	Total (*n* = 336)		AL-Nasser pediatric hospital (*n* = 175)		EL-Rantisi Pediatric hospital (*n* = 77)		Al-Durra pediatric hospital (*n* = 84)		*P* value
	100%		52%		23%		25%		
STANDARD (I): Every child receives evidence-based care and management of illness according to WHO guidelines.									
Extremely disagree	5	1.5%	1	0.6%	2	2.6%	2	2.4%	0.005
Disagree	30	8.9%	20	11.4%	2	2.6%	8	9.5%	
Don't know	77	22.9%	36	20.6%	23	29.9%	18	21.4%	
Agree	207	61.6%	102	58.3%	50	64.9%	55	65.5%	
Extremely agree	17	5.1%	16	9.1%	0	0.0%	1	1.2%	
STANDARD (II): The health information system ensures the collection, analysis and use of data to ensure early, appropriate action to improve the care of every child.									
Extremely disagree	10	3.0%	4	2.3%	2	2.6%	4	4.8%	0.002
Disagree	29	8.6%	14	8.0%	6	7.8%	9	10.7%	
Don't know	83	24.7%	51	29.1%	9	11.7%	23	27.4%	
Agree	161	47.9%	85	48.6%	36	46.8%	40	47.6%	
Extremely agree	53	15.8%	21	12.0%	24	31.2%	8	9.5%	
STANDARD (III): Every child with condition(s) that cannot be managed effectively with the available resources receives appropriate, timely referral, with seamless continuity of care.									
Extremely disagree	8	2.4%	1	0.6%	2	2.6%	5	6.0%	0.208
Disagree	20	6.0%	12	6.9%	3	3.9%	5	6.0%	
Don't know	89	26.5%	46	26.3%	18	23.4%	25	29.8%	
Agree	182	54.2%	97	55.4%	47	61.0%	38	45.2%	
Extremely agree	37	11.0%	19	10.9%	7	9.1%	11	13.1%	
STANDARD (IV): Communication with children and their families is effective, with meaningful participation, and responds to their needs and preferences.									
Extremely disagree	8	2.4%	3	1.7%	2	2.6%	3	3.6%	0.008
Disagree	23	6.8%	14	8.0%	3	3.9%	6	7.1%	
Don't know	72	21.4%	41	23.4%	12	15.6%	19	22.6%	
Agree	205	61.0%	94	53.7%	60	77.9%	51	60.7%	
Extremely agree	28	8.3%	23	13.1%	0	0.0%	5	6.0%	
STANDARD (V): Every child's rights are respected, protected and fulfilled at all times during care, without discrimination.									
Extremely disagree	5	1.5%	1	0.6%	2	2.6%	2	2.4%	0.547
Disagree	29	8.6%	16	9.1%	4	5.2%	9	10.7%	
Don't know	64	19.0%	34	19.4%	15	19.5%	15	17.9%	
Agree	186	55.4%	95	54.3%	41	53.2%	50	59.5%	
Extremely agree	52	15.5%	29	16.6%	15	19.5%	8	9.5%	
STANDARD (VI): All children and their families are provided with educational, emotional and psychosocial support that is sensitive to their needs and strengthens their capability.									
Extremely disagree	18	5.4%	10	5.7%	6	7.8%	2	2.4%	0.421
Disagree	25	7.4%	15	8.6%	3	3.9%	7	8.3%	
Don't know	75	22.3%	39	22.3%	13	16.9%	23	27.4%	
Agree	178	53.0%	93	53.1%	42	54.5%	43	51.2%	
Extremely agree	40	11.9%	18	10.3%	13	16.9%	9	10.7%	
STANDARD (VII): For every child, competent, motivated, empathic staff are consistently available to provide routine care and management of common childhood illnesses.									
Extremely disagree	8	2.4%	2	1.1%	2	2.6%	4	4.8%	0.034
Disagree	33	9.8%	22	12.6%	3	3.9%	8	9.5%	
Don't know	78	23.2%	39	22.3%	16	20.8%	23	27.4%	
Agree	167	49.7%	78	44.6%	48	62.3%	41	48.8%	
Extremely agree	50	14.9%	34	19.4%	8	10.4%	8	9.5%	
STANDARD (VIII): The health facility has an appropriate, child-friendly physical environment, with adequate water, sanitation, waste management, energy supply, medicines, medical supplies and equipment for routine care and management of common childhood illness.									
Extremely disagree	36	10.7%	21	12.0%	11	14.3%	4	5.2%	0.001
Disagree	165	49.1%	72	41.1%	38	49.4%	55	71.4%	
Don't know	107	31.8%	70	40.0%	25	32.5%	12	15.6%	
Agree	28	8.3%	12	6.9%	3	3.9%	13	16.9%	
Extremely agree	0	0.0%	0	0.0%	0	0.0%	0	0.0%	
Total scores of the eight standards (agree and extremely agree) of quality of care for children									
Agree and extremely agree	29.5%		29.1%		31.9%		28.4%		-

Continuous variables were expressed as means ± SD, while categorical variables were presented as percentages. The Chi-square test was used to examine differences in the prevalence of different categorical variable. *P* value ≤ 0.05 was considered as statistically significant.

Overall, the total scores of healthcare providers who agreed or strongly agreed with the standards were 29.5%; in addition, it was 29.1% for AL-Nasser Pediatric Hospital, 31.9% for EL-Rantisi Pediatric Hospital, and 28.4% for AL-Durra Pediatric Hospital.

**Figure 1:**
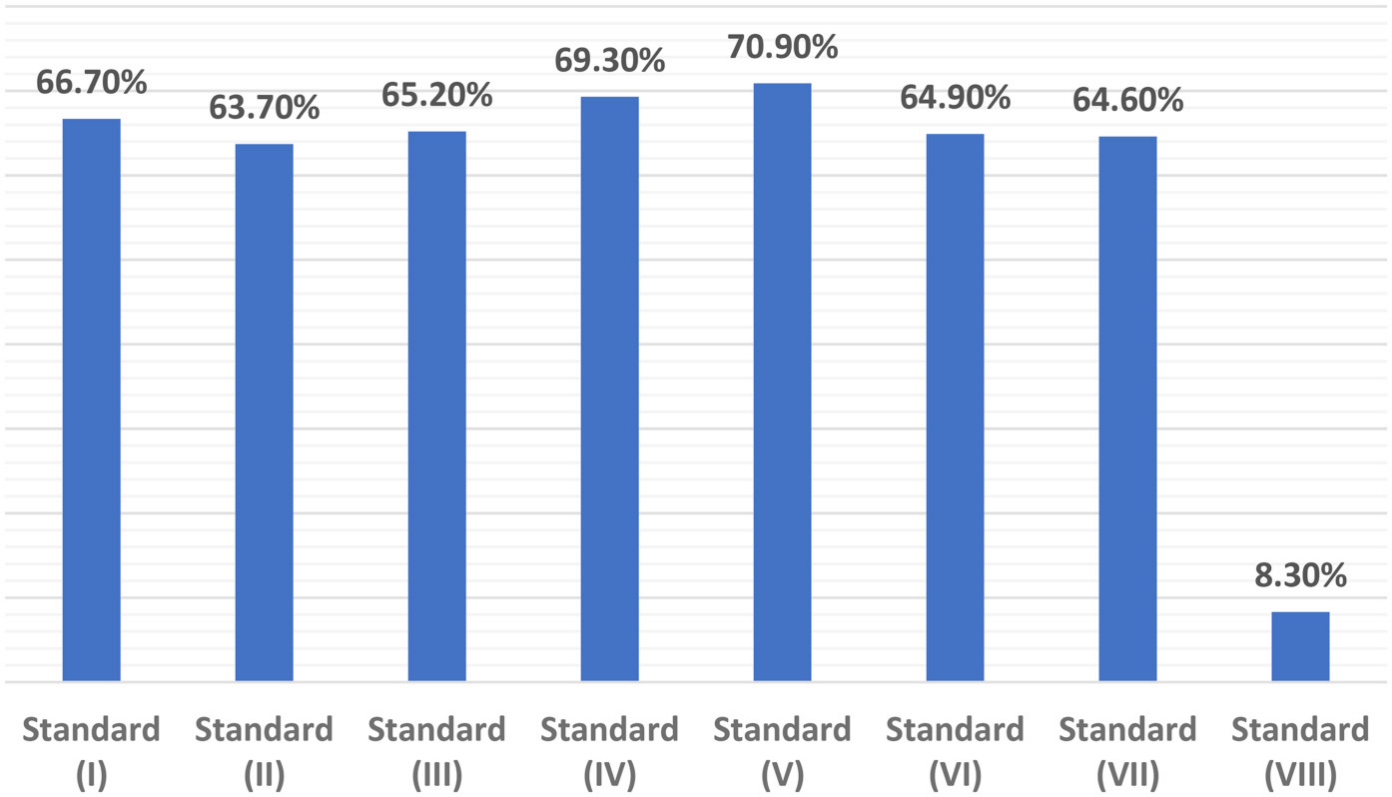
The distribution of the total scores for the healthcare providers (agree and strongly agree) for the included standards of the WHO quality of pediatric care integrated tool.

Figure 1 shows that the lowest percentage of healthcare providers (agree and strongly agree) for the included standards was for standard (VIII) at 8.3%, while the highest percentage was for standard (V) at 70.9%. The distribution of the total scores for the healthcare providers (agree and strongly agree) across the WHO quality of pediatric care integrated tool standards was as follows: 8.3% for standard (VIII), 63.7% for standard (II), 64.6% for standard (VII), 64.9% for standard (VI), 65.2% for standard (III), 66.7% for standard (I), 69.3% for standard (IV), and 70.9% for standard (V), all as confirmed by the study participants.

Table 3 presents the association between the overall total scores of health care providers' responses to the eight standards of quality care for children and the characteristics of the study participants. The findings revealed that health care providers aged 31–35 years were three times more likely to have better overall total scores compared to other age groups [Adjusted Odds Ratio = 3.014, 95% Confidence Interval = (1.255–7.241), *P* value = 0.014]. No significant associations were observed with any of the other variables.

**Table 3:** The association between the overall total scores of the health care provider's responses to the eight standards of quality of care for children with the characteristics of the study participants.

Variables	B (SE)	Adjusted Odds Ratio (95% CI)	Wald Statistics	*P*-value
Age				
20–25 years old		Ref		-
26–30 years old	0.107 (0.476)	1.113 (0.438–2.827)	0.051	0.822
31–35 years old	1.103 (0.447)	3.014 (1.255–7.241)	6.087	0.014^a^
36–40 years old	−0.619 (0.338)	0.539 (0.278–1.044)	3.358	0.067
>40 years old	−0.645 (0.344)	0.524 (0.267–1.030)	3.515	0.061
Gender				
Male		Ref		-
Female	−0.124 (0.245)	0.884 (0.546–1.429)	0.255	0.614
Qualification				
Diploma		Ref		-
Bachelor	−0.122 (0.609)	0.885 (0.268–2.919)	0.040	0.841
Master	−0.419 (0.525)	0.658 (0.235–1.840)	0.637	0.425
Ph.D.	−1.121 (0.582)	0.326 (0.104–1.019)	3.713	0.054
Special studies in pediatric care				
No		Ref		-
Yes	−0.383 (0.342)	0.682 (0.349–1.333)	1.252	0.263
Training courses in pediatric care				
No		Ref		-
Yes	−0.044 (0.242)	0.957 (0.595–1.539)	0.033	0.856
Years of experience				
<5 years		Ref		-
5–10 years	0.480 (0.344)	1.616 (0.823–3.174)	1.943	0.163
11–15 years	0.646 (0.361)	1.907 (0.941–3.866)	3.205	0.073
>15 years	−0.299 (0.308)	0.742 (0.405–1.357)	0.941	0.332
Years of experience in pediatric care				
<5 years		Ref		-
5–10 years	0.517 (0.288)	1.676 (0.954–2.946)	3.226	0.072
>10 years	0.541 (0.311)	1.717 (0.933–3.160)	3.016	0.082
Training duration weeks				
None		Ref		-
<5 weeks	−0.318 (0.453)	0.727 (0.299–1.768)	0.494	0.482
5–15 weeks	0.352 (0.533)	1.421 (0.500–4.044)	0.435	0.510
16–30 weeks	−0.318 (0.492)	0.727 (0.277–1.907)	0.419	0.517
31–45 weeks	−0.424 (0.524)	0.655 (0.235–1.827)	0.655	0.418
>45 weeks	1.822 (1.109)	6.182 (0.704–4.308)	2.699	0.214

Statistical testing using logistic regression. Ref, reference category; Adjusted odds ratio using 95% confidence interval in multinomial logistic regression analysis; CI, confidence interval; B, slop.

^a^ Difference is significant at the 0.05 level (2-tailed).

## 5 Discussion

The findings of this study provide valuable insights into the quality of pediatric care in the Gaza Strip, specifically in the three major governmental pediatric hospitals: Al-Nasr Pediatric Hospital, Al-Durra Pediatric Hospital, and Al-Rantisi Pediatric Hospital. Using the WHO integrated tool for assessing the quality of care, the results revealed suboptimal levels of healthcare provision, with the lowest scores noted for certain standards, particularly in the physical environment of the healthcare facilities. These results suggest a need for systemic improvements to ensure the quality of pediatric care, in line with WHO guidelines, which is essential for improving health outcomes for children.

Several studies have examined the quality of pediatric care in different regions, including resource-limited settings. A previous study found that healthcare facilities often struggled with providing high-quality care due to inadequate resources, poor physical infrastructure, and limited staff training, which are similar challenges identified in our study (13). Likewise, another study on the quality of pediatric care in public hospitals also revealed suboptimal performance in standards related to facility infrastructure, including water, sanitation, and waste management, similar to our findings regarding standard VIII (14). This emphasizes the universal need for improvements in physical infrastructure to provide an appropriate child-friendly environment.

In contrast, studies in more developed healthcare systems, such as those in Europe, show higher adherence to WHO standards, especially in areas like effective health information systems, timely referrals, and evidence-based care (15–17). These findings suggest that while the Gaza Strip faces significant challenges, particularly in terms of healthcare infrastructure, there are still areas where significant improvements can be made, especially by adopting strategies from successful healthcare models in developed countries.

For standard VIII: Physical environment, the results of this study highlight the stark need for improvement in the physical environment of pediatric hospitals. Only 8.3% of healthcare providers at the three hospitals agreed or strongly agreed with the statements related to having an appropriate, child-friendly environment. This is one of the lowest scores in the study, indicating that infrastructure challenges are a significant barrier to quality pediatric care. The lack of adequate water, sanitation, waste management, energy supply, medicines, and medical supplies not only affects day-to-day operations but also poses significant risks to patient safety and care quality. This is consistent with findings from other studies conducted in conflict zones or low-resource settings, where physical infrastructure issues are often a key limitation (18, 19).

For standard V: Rights of children, while the physical environment remains a challenge, the study found a relatively higher score for Standard V (70.9%), indicating that healthcare providers were relatively confident that children's rights were respected, protected, and fulfilled during care. This could be a result of training programs or policies that aim to ensure children's rights are upheld despite the challenging conditions. Nevertheless, while healthcare providers may have knowledge of child rights, the implementation of these rights in practice, particularly in the context of resource scarcity, remains a complex issue. A recent study in Palestine also emphasized the importance of children's rights within the healthcare setting, noting that while policy frameworks exist, challenges persist in their operationalization (20).

For standard II: Health information systems, the study showed that 63.7% of healthcare providers reported having adequate health information systems in place. This finding is consistent with research conducted in similar settings, where health information systems were found to be an important factor in ensuring timely and appropriate care (21). However, despite the relatively positive response regarding the use of health information systems, the differences observed between hospitals in the implementation of these systems underscore the variability in data management practices, which could impact decision-making processes and the overall quality of care.

For standard I: Evidence-based care, in terms of evidence-based care, 66.7% of participants reported adherence to WHO guidelines for illness management. While this figure is relatively high compared to other standards, it still reflects the need for ongoing training and the integration of evidence-based practices into routine pediatric care. Evidence from a similar study in Jordan (22) suggests that despite the presence of evidence-based guidelines, healthcare providers may face challenges in consistent implementation due to time constraints, resource limitations, and other external factors.

Finally, the findings of this study revealed that health care providers aged 31–35 years were significantly more likely to achieve higher overall total scores in their responses to the eight standards of quality care for children. One possible explanation is that this age group may be more actively engaged in continuous education, familiar with updated guidelines, and still adaptable to evolving best practices. In contrast, younger providers may lack sufficient clinical experience, while older providers may rely more heavily on past practices. However, it is also important to consider contextual factors such as institutional support, workload, and access to training opportunities, which may vary across settings.

## 6 Strength and limitations

The study's strengths include a comprehensive assessment of pediatric care quality in Gaza hospitals, a large sample size with a 94% response rate, and the use of standardized WHO tools for data collection. However, its limitations include the cross-sectional design, limiting the ability to assess trends or causal relationships, potential response bias, and limited generalizability to smaller or private healthcare facilities. Additionally, the study primarily focused on internal factors without fully exploring the external influences, such as political instability or external healthcare support.

## 7 Conclusion

This study highlights the gaps in the quality of pediatric care in the Gaza Strip, as perceived by healthcare providers, particularly in the domains of child-friendly environments, evidence-based care, and healthcare infrastructure. Despite the challenges, healthcare providers are committed to adhering to the WHO standards where feasible. It is imperative for stakeholders, including the Ministry of Health, international health organizations, and local policymakers, to focus on improving hospital infrastructure, ensuring better resource allocation, and enhancing healthcare providers' training to address the deficiencies highlighted in this study. The findings emphasize the need for a comprehensive approach to improving pediatric care, focusing on both the physical environment and the provision of adequate resources, alongside strengthening evidence-based practices and data-driven decision-making processes. The results serve as a crucial resource for future health policy and strategy development in the Gaza Strip.

## Data Availability

The raw data supporting the conclusions of this article will be made available by the authors, without undue reservation.
